# Biomedical named entity recognition using deep neural networks with contextual information

**DOI:** 10.1186/s12859-019-3321-4

**Published:** 2019-12-27

**Authors:** Hyejin Cho, Hyunju Lee

**Affiliations:** School of Electrical Engineering and Computer Science, Gwangju Institute of Science and Technology, 123 Chemdangwagi-ro, Buk-gu, Gwangju, Republic of Korea

**Keywords:** Text mining, Named entity recognition, Neural networks, Long short-term memory, Contextual information

## Abstract

**Background:**

In biomedical text mining, named entity recognition (NER) is an important task used to extract information from biomedical articles. Previously proposed methods for NER are dictionary- or rule-based methods and machine learning approaches. However, these traditional approaches are heavily reliant on large-scale dictionaries, target-specific rules, or well-constructed corpora. These methods to NER have been superseded by the deep learning-based approach that is independent of hand-crafted features. However, although such methods of NER employ additional conditional random fields (CRF) to capture important correlations between neighboring labels, they often do not incorporate all the contextual information from text into the deep learning layers.

**Results:**

We propose herein an NER system for biomedical entities by incorporating n-grams with bi-directional long short-term memory (BiLSTM) and CRF; this system is referred to as a contextual long short-term memory networks with CRF (CLSTM). We assess the CLSTM model on three corpora: the disease corpus of the National Center for Biotechnology Information (NCBI), the BioCreative II Gene Mention corpus (GM), and the BioCreative V Chemical Disease Relation corpus (CDR). Our framework was compared with several deep learning approaches, such as BiLSTM, BiLSTM with CRF, GRAM-CNN, and BERT. On the NCBI corpus, our model recorded an F-score of 85.68% for the NER of diseases, showing an improvement of 1.50% over previous methods. Moreover, although BERT used transfer learning by incorporating more than 2.5 billion words, our system showed similar performance with BERT with an F-scores of 81.44% for gene NER on the GM corpus and a outperformed F-score of 86.44% for the NER of chemicals and diseases on the CDR corpus. We conclude that our method significantly improves performance on biomedical NER tasks.

**Conclusion:**

The proposed approach is robust in recognizing biological entities in text.

## Background

With the increasing number of biomedical articles and resources, searching for and extracting valuable information has become challenging [1]. Researchers consider multiple information sources and transform unstructured text data into refined knowledge to facilitate research productivity [2, 3]. However, manual annotation and feature generation by biomedical experts are inefficient because they involve a complex process and require expensive and time-consuming labor [4]. Therefore, efficient and accurate natural language processing (NLP) techniques are becoming increasingly important for use in computational data analysis, and advanced text mining techniques are necessary to automatically analyze the biomedical literature and extract useful information from texts [5–8].

For extracting valuable information, such as relationships among objects, the identification of significant terms from texts is important. Meaningful terms or phrases in a domain, which can be distinguished from similar objects, are called named entities, and named entity recognition (NER) is one of the important tasks for automatically identifying these named entities in text and classifying them into pre-defined entity types [9, 10]. NER should be performed prior to tasks, such as relation extraction, because annotated mentions play an important role in research on text mining. In the biological domain, a fundamental task of biomedical NLP is the recognition of named entities, such as genes, diseases, chemicals, and drug names, from texts. However, biomedical NER is a particularly complex task because biological entities (i) continually increase with new discoveries, (ii) have large numbers of synonyms, (iii) are often referred to using abbreviations, (iv) are described by long phrases, and (v) are mixtures of letters, symbols, and punctuation [11, 12]. Several approaches have been proposed to solve these problems [1].

Most early methods for biomedical NER relied on dictionary- or rule-based approaches. NER systems using a dictionary-based method extract named entities in pre-defined dictionaries that consist of large collections of names for each entity type. Another NER system, using the rule-based approach, recognizes named entities by means of several rules that are manually defined based on their textual patterns [7, 9, 13]. The majority of these traditional approaches have shown significant improvements in terms of coverage and robustness, but rely heavily on a set of words in well-defined dictionaries and hand-crafted rules. Moreover, although relatively well-constructed dictionaries are available for common biological entities, such as disease and gene names, dictionaries for many other biological entities are not comprehensive or adequate [11]. In the case of rule-based methods, pre-defined patterns also depend on the specific textual properties of an entity class. In other words, entity-specific dictionaries and patterns require time-consuming processes and expert knowledge [7, 8].

To address the shortcomings of past approaches, traditional NER methods have been replaced by supervised machine learning methods, including hidden Markov models, maximum entropy Markov models, conditional random fields (CRFs), and the support vector machine [14–17]. Furthermore, machine learning methods are often combined with various others to yield hybrid approaches that are more accurate [18, 19]. Although most machine learning approaches have led to significant improvements in NER, and despite several general-purpose NER tools based on machine learning methods being available, they are still limited in terms of reliance on hand-crafted features and human labor for feature engineering [20–22].

Deep learning approaches using a large number of unstructured data items have lately drawn research interest and have been applied to NLP problems with considerable success. For NER tasks in the biomedical domain, a domain-independent method based on deep learning and statistical word embeddings, such as the bi-directional long short-term memory network (BiLSTM) with CRF and GRAM-CNN, has been shown to outperform state-of-the-art entity-specific NER tools such as a disease-specific NER tool DNorm and a chemical-specific NER tool ChemSpot [12, 18, 23–26]. Recently, Devlin et al. proposed a new architecture named BERT [27] for NLP. BERT (Bi-directional Encoder Representations from Transformers) is a deep bi-directional pre-trained self-attention model by the Transformer [28] and uses more than 2.5 billion words for pre-training the model and obtains new state-of-the-art results on various NLP tasks, including NER.

For machine learning, contextual information has already been demonstrated to lead to significant improvements [29]. Context representations usually define a collection of neighboring word embeddings in a window around the target word or an average of these window-based embeddings [30]. We propose herein an NER system designed to more explicitly deal with contextual information in text. The architecture of our system focuses on capturing important local contexts based on n-gram characters and word embeddings via BiLSTM and CRF. The performance of our model, Contextual LSTM with CRF (CLSTM), is evaluated using three biomedical corpora and various assessment methods.

## Results

### Data sources

#### Corpora

We used three kinds of corpora to train and test the NER models, where each contained manual annotations for one or more entity types. The corpora were the National Center for Biotechnology Information (NCBI) disease corpus for disease names [31], the BioCreative II Gene Mention (GM) corpus for gene names [32], and the BioCreative V Chemicals Disease Relationship (CDR) corpus for both disease and chemical names [33]. The corpora consist of a training set, a development set, and a test set, which were respectively used to construct the models, determine the optimal parameters for models, and evaluate the models. Table 1 lists the sizes of the corpora. We represented a sequence of labels in the IOB format (inside, outside, beginning), indicating that each token was at the beginning of an entity as a B-label, inside an entity as I-label, or outside it as an O-label. In this case, the labels simultaneously incorporated the type of named entity, such as disease or chemical, with the position of the token within the entity.

**Table 1:** Statistics of the NCBI, GM, and CDR corpora

Corpus	Entity	Unit	Training	Develop	Test	Total (Unit)
NCBI	Disease	Abstracts	592	100	100	792 (abstracts)
GM	Gene	Sentences	15000	-	5000	20000 (sentences)
CDR	Disease, Chemicals	Abstracts	500	500	500	1500 (abstracts)

**NCBI** We used the NCBI corpus for the disease NER task. The NCBI disease corpus is the gold standard of disease name recognition. It is a manually annotated resource for biomedical text created and curated by a team of 14 annotators. It consists of 793 PubMed abstracts and 6892 disease mentions, with 790 unique disease concepts mapped to MeSH and OMIM identifiers.

**GM** We used the GM corpus for the gene NER task. The second BioCreative challenge was held in 2006 and consisted of three tasks: gene mention, gene normalization, and protein–protein interaction. The entire corpus consisted of 20,000 sentences and a set of gene mentions and their alternative annotations judged by human annotators. This corpus did not contain a development set; hence, we randomly divided the training set into two parts to create the development corpus.

**CDR** We used the CDR corpus for the disease and chemical NER task. The BioCreative V challenge was organized for CDR tasks based on disease named entity recognition (DNER) and chemically induced disease (CID) relation extraction tasks. It is composed of 1500 articles with 4409 annotated chemical names, 5818 disease names, and 3116 CID relations. This corpus has become a valuable resource for research on text mining.

#### Parameters

Pretrained word embeddings are beneficial over random initializations in several NER tasks. Pyysalo et al. [34] trained the embedding model using approximately 23 million PubMed abstracts and nearly 700,000 PubMed Central full-text articles. We initialized our word representation using those trained by Pyysalo et al. We used 200 embedding dimensions with the skip-gram model at a window size of five [35]. These embeddings were fine-tuned during training. In experiments for BiLSTM, BiLSTM-CRF, and CLSTM, we used default values from Lample *et al* [24], except for three hyperparameters: (i) the tag scheme, which we set to the IOB scheme instead of IOBES; (ii) the number of dimensions of token embeddings and the size of the token LSTM hidden layer, which we set to 200 instead of 100; and (iii) pretrained embeddings, which we set to our embeddings instead of being none. For GRAM-CNN and BERT, we trained each model with its own default parameters.

### Evaluation

For comparative evaluation, we used BiLSTM without the CRF layer, BiLSTM-CRF [24], GRAM-CNN [12], and BERT [27]. For the comparison, we trained our CLSTM models on each corpus with one of three training options, called word-level model, character-level model, and word+char model. In the word- and character-level CLSTM models, window sizes at the word level or at the character level are needed, respectively. For the word+char CLSTM model, window sizes both at the character and word levels are required. To obtain the proper window size of each model, we used development sets. Odd numbers of window sizes, such as 3, 5, and 7, were used as candidate sizes to have equal context information for the left and right sides of the target word. Using the development sets, for the word-level CLSTM model, we decided on a window size of 5 for all three corpora. Similarly, for character-level CLSTM models, we decided on window sizes as 3, 5, and 7 for NCBI, GM, and CDR corpora, respectively. The word+char CLSTM model for NCBI used a window size of 5 for both the word and character levels, and the optional values for GM and CDR were set to 3 for both the word and character levels.

Using the test sets, we compared all methods in terms of precision, recall, and F-score. We performed strict matching at the IOB token level and strict and partial matching at the level of mention to compute these values. We counted the true positives (*TP*), false positives (*FP*), and false negatives (*FN*). The evaluation was based on measures of precision (*p*), recall (*r*), their harmonic average, and the F-score (*f*), as follows:

1 $$ p=\frac{TP}{TP+FP},\; r=\frac{TP}{TP+FN},\; f=\frac{2*p*r}{p+r}  $$

Table 2 shows the prediction performances over all corpora in terms of precision, recall, and F-score using three evaluation methods (i.e. the strict matching, the partial matching, and the IOB tag matching). The first four rows in each table show the performance of other methods, while the last three rows show the results of the CLSTM models. F-scores of CLSTM that outperformed the comparative methods are marked in bold.

**Table 2:** Comparison of performance for comparative methods on the NCBI, GM, and CDR corpora using strict and partial matching and IOB tag matching

Model		p	r	f	p	r	f	p	r	f
**Strict matching**		NCBI			GM			CDR		
BiLSTM		78.91	82.60	80.71	72.22	72.44	72.33	83.56	80.26	81.88
BiLSTM-CRF		82.19	84.58	83.37	80.79	79.81	80.30	87.52	83.58	85.50
GRAM-CNN		84.45	83.92	84.18	80.23	78.83	79.53	86.08	85.49	85.79
BERT		81.07	80.73	80.90	81.72	81.59	**81.65**	86.21	85.23	85.72
CLSTM	word level	85.94	84.69	**85.31**	81.00	80.77	80.89	87.23	85.51	**86.36**
	character level	85.40	84.06	**84.72**	81.09	80.38	80.73	87.19	84.69	**85.92**
	word+char levels	84.73	86.67	**85.68**	81.75	81.14	81.44	87.25	85.66	**86.44**
**Partial matching**		NCBI			GM			CDR		
BiLSTM		86.67	90.73	88.65	87.98	88.25	88.11	91.14	87.54	89.30
BiLSTM-CRF		91.19	93.85	92.51	93.18	92.04	92.61	94.27	90.00	92.08
GRAM-CNN		94.36	93.78	**94.07**	93.09	91.47	92.27	92.47	91.83	92.15
BERT		88.39	88.02	88.20	92.65	92.51	92.58	91.82	90.77	91.29
CLSTM	word level	93.66	92.29	92.97	92.81	92.54	**92.67**	93.60	91.74	**92.66**
	character level	93.76	92.29	93.02	93.05	92.25	**92.65**	93.42	91.59	**92.49**
	word+char levels	93.71	93.13	93.42	93.35	92.65	**93.00**	93.48	91.77	**92.62**
**IOB tag matching**		NCBI			GM			CDR		
BiLSTM		84.56	88.03	86.26	84.23	81.48	82.83	89.81	78.68	83.87
BiLSTM-CRF		84.13	88.32	86.18	88.34	84.47	86.36	90.54	81.34	85.69
GRAM-CNN		88.73	86.59	87.65	87.75	84.09	85.89	89.72	83.03	86.24
BERT		88.42	83.15	85.70	89.50	86.26	**87.85**	88.69	85.01	86.81
CLSTM	word level	89.18	89.01	**89.10**	88.99	84.89	86.89	89.99	83.19	86.45
	character level	88.21	88.47	**88.34**	87.72	85.21	86.45	89.95	83.19	86.43
	word+char levels	89.98	87.74	**88.84**	87.13	86.88	87.00	90.56	83.41	**86.83**

**Strict matching** When start and end boundaries and the type of a predicted mention and those of a gold standard mention are identical, it is considered correct prediction. This evaluation criterion evaluates tag units as one result that recognizes mentions from the B-tag to its end. On the NCBI corpus, our model with word+char levels attained an F-score of 85.68%, which is a 1.5% improvement over the previous methods. Among the previous methods, GRAM-CNN achieved the best F-score of 84.18%. Moreover, word-level CLSTM and character-level CLSTM also obtained results (85.31% and 84.72%, respectively) better than those of the comparative approaches. On the GM corpus for the gene NER, our CLSTM yielded an F-score of 81.44%. Although BERT improved the F-score by 0.21% compared with CLSTM (81.65% vs. 81.44%, respectively), this difference was slight considering that BERT incorporates other huge datasets as well as the GM corpus. On the CDR corpus, the word+char levels CLSTM model had an F-score of 86.44% for chemicals and disease NER. As for the CDR corpus, when we assessed the results using the strict matching, all results of the CLSTM with word and character and word+char levels outperformed those of the previous method (86.36%, 85.92% and 86.44%, respectively).

**Partial matching** When start and end boundaries of a predicted mention and those of a gold standard mention are overlapping, and types of the prediction and the gold standard are the same, and it is considered correct prediction. When this evaluation criterion was used, all models yielded F-scores higher than those obtained using other evaluation criteria (i.e., strict and IOB tag matchings). Although our model recorded a slightly inferior performance to GRAM-CNN on the NCBI corpus, our NER model achieved the best F-scores for GM and CDR corpora. Among previous methods, BiLSTM-CRF and GRAM-CNN achieved the best F-score on the GM and CDR corpora, respectively.

**IOB tag matching** We further assessed the performance of our method on the three corpora at the level of tokens. For each IOB tag, the agreement between prediction and the gold standard tag is assessed. This procedure involves comparing the results of the gold standard tags with those of the predicted tags at the token level. This evaluation depends on the lengths of the mentions. On the NCBI corpus, our model with word-level layers attained an F-score of 89.10%, which shows a 1.45% improvement over the previous methods. Among the previous methods, GRAM-CNN achieved the best F-score (87.65%). Moreover, all results of the CLSTM (88.34% and 88.84% for the character-level CLSTM and word+char levels CLSTM, respectively) outperformed other approaches. Similar to the strict matching, the BERT model on the GM corpus improved the F-score compared with the proposed model. On the CDR corpus, the word+char levels CLSTM model represents a maximum F-score of 86.83%, which improves the F-score by 0.02% compared with the previous method (86.83% vs. 86.81%). From Table 2, all results of the CLSTM on the GM and CDR corpus outperformed those of the previous methods, except for BERT.

### Model robustness

Character vectors were randomly initialized for every character, and word vectors that do not have an embedding in the lookup table were mapped to a UNK embedding before being entered into the model [24]. Therefore, the performance of our models might depend on the random initialization of weights. Thus, we independently trained the CLSTM model five times and analyzed the results by applying strict matching to estimate the robustness of our models with respect to initialization.

Table 3 shows the performance comparison between our CLSTM model for all five trials and the other methods on the NCBI, GM, and CDR corpora. For each method and corpus, we used optimal hyperparameters obtained from development sets. In the NCBI corpus, although the best score of the other methods yielded an F-score of 84.18%, our model achieved the best F-score of 85.68% and the worst F-score of 85.02%. Thus, the worst performance of the CLSTM model was better than that of GRAM-CNN with a difference of 0.84%. Despite recording a slightly inferior performance compared with BERT on the GM corpora, our NER model was better than all other comparative models. In the CDR corpus, our models also outperformed all other methods, which were similar with the performance on the NCBI corpus. Therefore, the results confirm the superiority of our model, regardless of the randomness of initialization.

**Table 3:** Comparison between a series of CLSTM (contextual long short-term memory networks [LSTMs] with conditional random fields [CRF]) experiments and the comparative methods on the NCBI, GM, and CDR corpora using strict matching

Strict matching		NCBI			GM			CDR		
Model	Trial #	p	r	f	p	r	f	p	r	f
BiLSTM	-	78.91	82.60	80.71	72.22	72.44	72.33	83.56	80.26	81.88
BiLSTM-CRF	-	82.19	84.58	83.37	80.79	79.81	80.30	87.52	83.58	85.50
GRAM-CNN	-	84.45	83.92	84.18	80.23	78.83	79.53	86.08	85.49	85.79
BERT	-	81.07	80.73	80.90	81.72	81.59	**81.65**	86.21	85.23	85.72
CLSTM (word+char levels)	1	84.73	86.67	**85.68**	81.75	81.14	81.44	87.25	85.66	**86.44**
	2	84.43	85.83	**85.12**	81.26	80.67	80.96	87.16	85.40	**86.27**
	3	86.18	84.48	**85.32**	82.07	80.24	81.14	87.93	84.56	**86.21**
	4	85.56	85.21	**85.39**	82.97	79.66	81.28	87.71	85.17	**86.42**
	5	84.62	85.42	**85.02**	81.02	80.70	80.86	88.27	84.36	**86.27**
CLSTM average		85.10	85.52	**85.31**	81.81	80.48	81.14	87.66	85.03	**86.33**

## Discussion

### Error analysis

We analyzed error cases on the test corpora and classified them into several cases as follows:

- The entity boundary is not clear due to adjective phrases: For example, our model annotated “female breast cancer” and “idiopathic hemolytic uremic syndrome” as disease entities. However, disease mentions in the NCBI test set were “breast cancer” and “hemolytic uremic syndrome”, respectively. On the other hand, although disease mentions in the NCBI test set were “non-inherited breast carcinomas”, “sporadic T-cell leukaemia”, and “dominantly inherited neurodegeneration”, our model predicted “breast carcinomas”, “T-cell leukaemia”, and “neurodegeneration”, respectively.
- Elliptical coordinated compound noun phrases are used: This case is a kind of coordinate structures, where two or more words of the same type are combined into a larger phrase with the same semantic relation [36,37]. For example, names such as “pineal tumours and retinal tumours” and “colorectal adenomas and/or colorectal carcinoma” are often described in biomedical abstracts as “pineal and retinal tumours” and “colorectal adenomas and/or carcinoma” to avoid word repetition. Moreover, they were annotated as a single entity in the NCBI test set. For these cases, our model predicted their entity boundaries as “tumours” in the first example, and “colorectal adenomas” and “carcinoma”, respectively, in the second example.
- Entity contains brackets: This case often happens when an entity name and its acronym appear together with brackets. For example, “62-kDa protein (p62)”, which contains a gene name, its acronym “p62” and brackets, was annotated as a single gene mention in the GM test set. However, CLSTM separately predicted gene mentions as “62-kDa protein” and “p62” without brackets.
- Different entity types are predicted: When an entity type is nested in another entity type, the different entity type was predicted. This is more likely to happen when multiple entity types are predicted at the same time. For example, “serotonin syndrome” in the CDR test set was annotated as a disease mention. However, our model predicted “serotonin” as a chemical entity. Another example is that although “hepatitis B surface antigen” was annotated as a chemical type, our model predicted “hepatitis B” as a disease type.
- The corpus annotation inconsistency: The same disease was annotated differently in the same corpus. For example, “type I autosomal dominant cerebellar ataxia” was annotated as a disease mention in the NCBI corpus (PubMed ID: 7573040). However, in “Eye movement abnormalities correlate with genotype in autosomal dominant cerebellar ataxia type I (PubMed ID: 9506545),” only “cerebellar ataxia type I” was annotated as a disease mention, and did not include “autosomal dominant.” In the latter case (PubMed ID: 9506545), our model predicted “autosomal dominant cerebellar ataxia type I.”

The above analysis shows that some NER errors occurred due to various forms of entity mentions, and usually occurred in entity boundaries. For example, when we mannally examined false positives on the NCBI corpus, we found that 35.3% and 9.3% of NER errors were due to entity boundaries and elliptical coordination errors, respectively. Thus, it is important to develop the NER model to resolve these ambiguities.

### Cross-corpus evaluation

We performed cross-corpus evaluation between the NCBI and the CDR corpora. We tested the disease entities in the CDR corpus using the model trained on the NCBI disease corpus, and also tested mentions in the NCBI disease corpus using the model trained on the CDR corpus.

Table 4 shows that our CLSTM model had a higher F-score than those of other models except BERT. Although the precision of the CLSTM model was higher than that of BERT, BERT had higher recall values and F-scores. The high recall values may be because BERT has already been pre-trained with huge volumes of data from general datasets. Thus, although the guidelines for constructing two corpora of disease mentions (NCBI and CDR corpora) are different in terms of determining disease mentions, BERT can have a high recall value. For constructing each corpus, the authors of NCBI used the 2012 version of MEDIC, which integrated both OMIM and MeSH disease terms. On the other hand, the authors of CDR used the 2015 version of MeSH terms and annotated disease mentions with a ‘-1’ identifier (ID), even if the ID mapping for disease mentions is not possible. For example, although “pain” and “necrosis” in the CDR corpus were treated as disease mentions with “D010146” and “D009336”, respectively, these words were not annotated in the NCBI corpus. To examine the difference between two corpora, we counted disease mentions annotated in the NCBI test data, but not annotated as disease mentions in the CDR training data despite appearing in the sentences of the CDR corpus, and vice versa. We found 19 such mentions out of 960 mentions in the NCBI corpus, and 83 out of 10,875 mentions in the CDR corpus. Although our model correctly predicted 4 and 7 mentions in each corpus, BERT correctly predicted 11 and 41 mentions for NCBI and CDR corpus, respectively. It implies that the CLSTM model is more likely to reflect characteristics of the training data than BERT. Thus, even though our model may have lower recall values than BERT, it demonstrated higher precision. Note that as each corpus had different optimal window sizes from development sets, we tried several window sizes in Table 4.

**Table 4:** Comparison of the performance of cross-corpus evaluation for comparative methods using strict matching

Strict matching		train CDR → test NCBI^a^			train NCBI → test CDR^b^		
Model		p	r	f	p	r	f
BiLSTM		57.32	37.92	45.64	55.19	30.79	39.52
BiLSTM-CRF		68.34	36.88	47.90	58.30	38.74	46.55
GRAM-CNN		59.74	42.81	49.88	58.48	33.21	42.36
BERT		68.92	53.13	**60.00**	54.17	61.44	**57.57**
CLSTM	word level	62.42	48.96	54.87	60.92	38.09	46.87
	character level (3)^c^	68.12	44.06	53.51	**62.74**	32.66	42.96
	character level (7)^c^	65.08	45.63	53.64	60.69	21.75	32.02
	word+char levels (3, 3)^d^	66.77	43.75	52.86	54.00	44.08	48.54
	word+char levels (5, 5)^d^	**69.36**	42.92	53.02	57.63	39.51	46.88

^a^Test the disease entities in the NCBI corpus using the model trained on the CDR corpus

^b^Test the disease entities in the CDR corpus using the model trained on the NCBI corpus

^c^The number in parentheses represents the window size at the character level.

^d^The numbers in parentheses represent the window sizes at the word and character level, respectively

### Computational time

We measured computational time for CLSTM and for the comparative models. We ran all models in a hexa-core workstation using an i7-5930K CPU and a Titan Xp GPU with 12G memory and set a default training epoch of 100 on each dataset. Table 5 shows the training time of each model on three datasets. Overall, the execution time was determined in proportion to the size of the data. The fastest method was BERT, followed by BiLSTM, and GRAM-CNN had the longest training time. We observed that the character-level CLSTM model had relatively faster training time than other CLSTM models because the character embedding dimension of CLSTM was smaller than the word and word+char embedding dimensions of CLSTM. However, the CLSTM model required 20% longer training times than the BiLSTM-based models. The reason for the superior speed of BERT compared with the other methods is that BERT is a fine-tuning system and does not require the training of a deep neural network from scratch. However, the original pre-training of BERT took 4 days [27].

**Table 5:** Comparison of training time between CLSTM (contextual long short-term memory networks [LSTMs] with conditional random fields [CRF])and comparative methods for the NCBI, GM, and CDR corpora

Training time (Hours)		NCBI	GM	CDR
BiLSTM		4.08	10.77	4.39
BiLSTM-CRF		4.70	12.56	5.23
GRAM-CNN		11.27	34.08	12.64
BERT		1.01	10.04	3.72
CLSTM	word level	5.54	14.57	5.98
	character level	4.84	13.30	5.64
	word+char levels	5.84	14.73	5.91
	Average	5.41	14.20	5.84

## Conclusions

In this study, we investigated neural architectures with contextual information for biomedical named entity recognition based on various corpora and word embeddings. The experimental results show that our system outperforms several other NER approaches and exhibits similar performance to the transfer learning approach. The results of this study will help to make biomedical text mining more accurate and more robust irrespective of the entity type.

## Methods

### CLSTM

This section provides a brief description of the architecture of our CLSTM model. We provide details of the model from scratch.

#### LSTM

Recurrent neural networks (RNNs) are specially designed to process sequential data. They represent connections between previously occurring hidden states and a given hidden state, and thus reflect the network’s historical information. While the RNN is a simple and powerful model in theory, it cannot capture long-term dependencies because of problems of vanishing and exploding gradients, where the gradients may exponentially decline and grow over long sequences [38*–*40].

Long short-term memory networks (LSTMs) [41] are variants of the RNN applied to a memory cell to learn long-term dependencies. An LSTM unit is composed of three gates: an input gate, a forget gate, and an output gate. These gates control the amount of information for the network to remember and forget for the next time step.

In sequence-labeling tasks like NER, determining the contexts in sentences, where both past and future contexts are useful, is important. However, standard LSTMs can use only previous contexts without future information. Graves et al. [42] introduced a BiLSTM model, the basic idea of which is to describe each sequence in the forward and reverse directions to two separate layers. Two hidden states, $\overrightarrow {h}$ and $\overleftarrow {h}$, are then concatenated to represent the final output. For an input sentence (*x*_1_,*x*_2_,…,*x*_*n*_) containing *n* words, an LSTM computes a left representation $\overrightarrow {h_{t}}$ of the given sentence at every word *t*. Similarly, a representation of the right context $\overleftarrow {h_{t}}$ can be achieved from the same sequence in reverse. As a result, BiLSTM yields the representation of a word by concatenating the outputs of its left and right contexts, $h_{t}=[\overrightarrow {h_{t}}, \overleftarrow {h_{t}}]$ [23,40,43].

#### CRF

NER can be considered a sequence-labeling problem, which means that words in a given sentence are tokens to be assigned proper labels. For sequence-labeling tasks, considering correlations represented by the best joint probability between adjacent labels and the entire sequence of labels is beneficial. Therefore, we jointly decode label sequences using a CRF layer instead of independently modeling tagging decisions [8,20,21,44].

Formally, we use **x**=(*x*_1_,*x*_2_,…,*x*_*n*_) to represent an input sequence, where *x*_*i*_ is the input vector of the *i*-th word, and **y**=(*y*_1_,*y*_2_,…,*y*_*n*_) represents a sequence of predicted labels for input **x**. All components *y*_*i*_ of **y** are assumed to range over a set *L*(**x**), which is a possible labeling sequence for **x**. The global feature of CRF, **F**(**y**,**x**), is the summation of CRF’s local feature vector **f**(**y**,**x**,*i*) for input sequence **x** and label sequence **y**, where *i* ranges over input positions. The probabilistic model for the CRF defines a conditional probability *p*(**y**|**x**,**λ**) over all possible sequences of labels **y**, given **x** and weight vector *λ* in the following form:

2 $$ p\left(\mathbf{y} \vert \mathbf{x},\mathbf{\lambda} \right)=\frac{1}{Z\left(\mathbf{x} \right)} \text{exp} \left(\mathbf{\lambda} \cdot \mathbf{{F}} \left(\mathbf{y}, \mathbf{x} \right) \right),  $$

where $Z\left (\mathbf {x} \right) = {\sum \nolimits }_{\mathbf {y}'\in L\left (x \right)} {\text {exp} \left (\mathbf {\lambda } \cdot \mathbf {{F}} \left (\mathbf {y}', \mathbf {x} \right) \right)}$ is a normalization factor.

#### N-gram

In linguistics, an n-gram is a sub-sequence of *n* contiguous items extracted from a given text. Although the items can be of various types, such as characters and words in text as well as base pairs of DNA sequences and amino acids of protein sequences, we consider herein only the text data of natural language processing. Character-level n-grams represent n-character slices of a word, while word-level n-grams represent n-word slices of a sentence. For example, word-level bi-grams (*n*=2) in the phrase “biomedical named entity recognition” are “biomedical named,” “named entity,” and “entity recognition.” Similarly, character-level tri-grams (*n*=3) in the word “disease” are “dis,” “ise,” “sea,” “eas,” and “ase.” N-gram models are robust at statistically modeling language and at natural text processing without relying on language-specific resources [45,46].

#### CLSTM

**Fig. 1:**
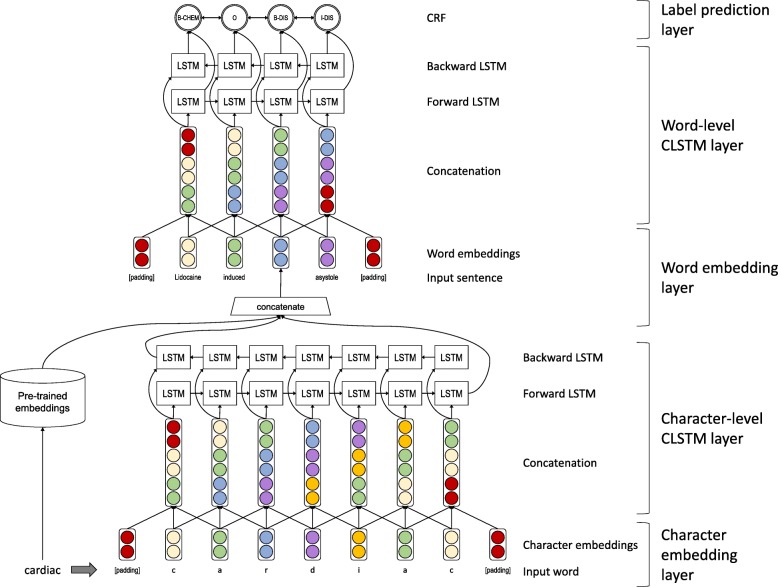
Pipeline of the CLSTM (contextual long short-term memory networks [LSTMs] with conditional random fields [CRF])model

To utilize contextual information in several NLP tasks, neural network-based algorithms that incorporate a large amount of unlabeled data [47*–*49] and neural network models such as BiLSTM that capture contextual information in an input text have been developed. In this study, to utilize more contextual information contained in sentences, we introduce the contextual long short-term memory networks with the CRF (CLSTM) model, which maximizes benefits of BiLSTM-CRF [24] and n-gram models for contextual information. While BiLSTM represents a certain target word or a character using an input vector of itself, CLSTM represents it by concatenating input vectors of its neighbors and itself. Figure 1 shows the architecture of the CLSTM model, which has the following major components: (i) a character-embedding layer, where each character in an input text is mapped to a character embedding; (ii) a character-level CLSTM layer, where character embedding vectors are input and character embedding vectors are output with the output character vector created by concatenating its left and right character embeddings within a pre-defined window size; (iii) a word-embedding layer in which each word in an input text is mapped to a word vector composed of concatenation of pretrained word vectors and the character-level representation; (iv) a word-level CLSTM layer that uses word vectors as input and output and, in a similar manner to the character level, the output is formed by concatenating its left and right word embeddings within a pre-defined window size; and (v) a label prediction layer in which for each word in the input text, the final CRF layer predicts proper entity labels based on the sequence of probabilities.

For the word-level layers, we split a sentence into words by white spaces and punctuation marks such as commas and hyphens. An input sentence *S* consisting of split words *w* is represented as *S*=[*w*_1_,*w*_2_,...,*w*_*n*_]. By representing *w*_*i*:*i*+*j*_=*w*_*i*_⊕*w*_*i*+1_⊕...⊕*w*_*i*+*j*_, where ⊕ is the concatenation symbol, {*w*_1:*d*_,*w*_2:*d*+1_,...,*w*_*n*−*d*+1:*n*_} is then used as the input of the word-level layer for the window size *d*. However, it cannot be well defined for words near the beginning and the end of the word. Therefore, we augment these embeddings to deal with the border effect [50]. We concatenate ⌊*d*/2⌋ paddings to the beginning and the end of the input of the CLSTM layer. For example, when the window size *c* is 3, the length of the word with paddings becomes *n*+(⌊*d*/2⌋∗2)=*n*+2, and a new input is given as *S*_*new*_=[*w*_0:2_,*w*_1:3_,*w*_2:4_,...,*w*_*n*−2:*n*_,*w*_*n*−1:*n*+1_], where *w*_0_ and *w*_*n*+1_ are paddings. This summarizes the contextual information of words in the input text. Similarly, the character-level representation of each word is computed by the CLSTM layers using character embeddings.

The CLSTM memory cell at the time step *t* is implemented as follows:

3 $$ x_{t^{'}} \,=\, x_{t-\lfloor d/2 \rfloor} \!\oplus... \oplus x_{t-1} \!\oplus x_{t} \oplus x_{t+1} \oplus... \oplus x_{t+\lfloor d/2 \rfloor},  $$

4 $$ i_{t} =\sigma \left(W_{xi}x_{t^{'}} + W_{hi}h_{t-1} + W_{ci}c_{t-1} + b_{i} \right),  $$

5 $$ c_{t} \,=\, \left(1-i_{t} \right) \odot c_{t-1} + i_{t} \odot \text{tanh}\left (W_{xc}x_{t^{'}} + W_{hc}h_{t-1} + b_{c} \right),  $$

6 $$ o_{t} = \sigma \left(W_{xo}x_{t^{'}} + W_{ho}h_{t-1} + W_{co}c_{t-1} + b_{o} \right),  $$

7 $$ h_{t} = o_{t} \odot \text{tanh}\left(c_{t}\right),  $$

where $\phantom {\dot {i}\!}x_{t^{\prime }}$ is the concatenation of character embeddings and the concatenation of word embeddings for the character-level CLSTM layer and word-level CLSTM layer, respectively; *d* is the pre-defined window size; *h*_*t*_ is the hidden state at time *t*; *W* is the weight matrix; *b* is the bias vector; *σ* is the sigmoid function; tanh is the hyperbolic tangent function; and the ⊙ operation denotes element-wise multiplication. We apply herein a variation of the LSTM unit to use coupled input and forget gates [24].

Finally, the output vectors of CLSTM layers are fed to the CRF layer to jointly decode the best label sequence. For the CRF layer, we use a state transition matrix to predict the tag at any given time. We denote by *T* a transition matrix and *T*_*i,j*_ a transition score from the *i*-th tag to the *j*-th tag. For a given sentence **x**=(*x*_1_,*x*_2_,…,*x*_*n*_), we denote by *P* the score matrix of the outputs of the CLSTM hidden layers. The *P*_*i,j*_ represents the score of the *j*-th tag at the result of the *i*-th word in the given sentence **x**. For a sequence of predicted labels **y**=(*y*_1_,*y*_2_,…,*y*_*n*_), the sum of scores from the LSTM networks along with the transition scores gives the final score of the sentence **x** and a sequence of predictions **y**. The final score can be expressed as follows:

8 $$ s\left(\mathbf{x},\mathbf{y}\right)=\sum\limits_{i=0}^{n}T_{y_{i},y_{i+1}}+\sum\limits_{i=1}^{n}P_{i,y_{i}}  $$

where *y*_0_ and *y*_*n*+1_ are the start and end tags of a sentence, respectively [24].

## Data Availability

All datasets used in this study are publicly available on http://gcancer.org/clstmdata.

## Abbreviations

BERT: Bi-directional encoder representations from transformers
BiLSTM: Bi-directional long short-term memory network
CDR: BioCreative V chemical disease relation corpus
CID: Chemical-induced disease
CLSTM: Contextual long short-term memory networks with CRF
CRF: Conditional random fields
DNER: Disease named entity recognition
f: F-score
FN: False negative
FP: False positive
GM: BioCreative II gene mention corpus
LSTM: Long short-term memory network
NCBI: National Center for Biotechnology Information disease corpus
NER: Named entity recognition
NLP: Natural language processing
p: precision
r: recall
RNN: Recurrent neural network
TP: True positive
